# ﻿Mycobiont-specific primers for lichenized fungal genus *Cladonia* (Cladoniaceae, Ascomycota)

**DOI:** 10.3897/mycokeys.127.167091

**Published:** 2026-01-06

**Authors:** Maia Biwersi, Maonian Xu, Starri Heiðmarsson, Snæbjörn Pálsson, John L. Sorensen, Elín S. Ólafsdóttir

**Affiliations:** 1 Faculty of Pharmaceutical Sciences, University of Iceland, Hofsvallagata 53, IS-107 Reykjavik, Iceland; 2 Northwest Iceland Nature Research Centre, Aðalgata 2, IS-550 Sauðárkrókur, Iceland; 3 Faculty of Life and Environmental Sciences, University of Iceland, Sturlugata 7, IS-102 Reykjavik, Iceland; 4 Department of Chemistry, University of Manitoba, Winnipeg, Manitoba, R3T 2N2, Canada

**Keywords:** *
Cladonia
*, Cladoniaceae, *MCM7*, mtSSU, nrITS, PCR, primer design, *RPB2*

## Abstract

Phylogenetic studies in lichenized fungi need reliable primers, as off-target amplification of environmental fungi or the lichen photobiont presents a challenge. In this study, new mycobiont-specific primers were developed and evaluated for four genetic markers (nrITS, *RPB2*, *MCM7*, and mtSSU) within the diverse genus *Cladonia*. A dataset of 110 specimens was used to assess amplification success, sequence quality, and taxonomic resolution. All markers showed high PCR success rates, with nrITS, *MCM7*, and mtSSU primer pairs exceeding 90%. nrITS displayed the highest sequence variability and nucleotide diversity, whereas mtSSU was the most conserved. Pairwise genetic distance analyses revealed that nrITS provided the strongest species-level resolution, *RPB2* offered intermediate divergence, *MCM7* was informative at the clade level but limited for closely related subclades, and mtSSU was best suited for deeper phylogenetic splits. This study shows the value of targeted primer design, and the new primers proved to be robust tools for future molecular identification and evolutionary research in *Cladonia*.

## ﻿Introduction

Lichens are symbiotic consortia of fungi and photosynthesizing partners (e.g., green algae and cyanobacteria), exhibiting adaptability to extreme environmental conditions (Allen and Lendemer 2022). They are ubiquitous in all terrestrial environments and produce over 1,000 different natural products (Calcott et al. 2018). *Cladonia* is a genus of lichen-forming fungi within the Cladoniaceae family, a widely distributed family of lichenized fungi found across the globe. Extensive phylogenetic studies have confirmed the monophyly of the family and the relationships among the genera within it (Stenroos et al. 2002, 2019; Myllys et al. 2002; Beiggi and Piercey-Normore 2007). Cladonia consists of 13 subgenus lineages and a diversity of 475 accepted species and is recognized as Cladoniaceae’s largest genus (Stenroos et al. 2019). However, because many morphological traits are shared across species—potentially due to parallel evolution—resolving the evolutionary history of *Cladonia* will require broader and more diverse taxon sampling (Osyczka 2006; Stenroos et al. 2019).

Molecular markers with suitable mutation rates and specific primers are key to resolving evolutionary relationships in lichens (Amit Roy 2014). Amplifying genetic markers in lichens presents various challenges and can significantly affect the success of polymerase chain reactions (PCR) (Zhou and Stanosz 2001; Xu et al. 2023). DNA extractions often co-isolate algal or microbial DNA, thalli may contain multiple *Cladonia* species, and lichen secondary metabolites can inhibit PCR efficiency (Cubero et al. 1999; Muggia et al. 2009; Kelly et al. 2011). In addition, primer binding sites are not always conserved across lineages, leading to mismatches and uneven amplification success (Xu et al. 2023).

For barcoding of species within the genus *Cladonia*, it has been recommended to combine two genetic markers (Pino-Bodas et al. 2013): the nuclear ribosomal internal transcribed spacer (nrITS) and the RNA polymerase II second largest subunit (RPB2) (Schoch et al. 2012; Farkas et al. 2024). Among protein-coding markers, *RPB2* shows the highest correct identification percentage but is difficult to amplify with available primers (Yahr et al. 2006; Pino-Bodas et al. 2013; Stenroos et al. 2019), and thus new primers need to be designed. Other markers, such as mitochondrial small subunit ribosomal DNA (mtSSU) and nuclear minichromosome maintenance complex protein 7 (*MCM7*), are commonly used in phylogenetic studies due to their evolutionary significance and variability across taxa (Raja et al. 2011; Heiðmarsson et al. 2017).

The selection of nrITS and *RPB2* was based on their combined power for *Cladonia* specimen identification (Pino-Bodas et al. 2013). *MCM7* and mtSSU were included because they are commonly used in other lichen taxa while largely unexplored in *Cladonia* (Zoller et al. 1999; Schmitt et al. 2009). In this study, we designed and tested mycobiont-specific primers across a broad sampling of *Cladonia* taxa, aiming for high specificity to *Cladonia* mycobiont sequences while avoiding amplification of non-lichen-forming ascomycetes and basidiomycetes.

## ﻿Materials and methods

### ﻿Primer design

All primers were designed through visual inspections of sequence alignments, without the use of automated software tools. The nomenclature of newly designed primers follows the recommendations proposed by Gargas and DePriest (1996). Details on the primer sequences, binding locations, and predicted melting temperatures (Tm) are provided in Table 1.

**Table 1.** List of primers tested for *Cladonia* species with corresponding locations within the loci mtSSU, *MCM7*, *RPB2*, and nuclear rRNA loci (nuSSU and nuLSU) covering the variable internal spacer region (nrITS), sequence, and melting temperature.

Primer	Location	Sequence 5´-3´	Tm [°C]
mtSSU-581-5’	581–600	GGAGGAATGTATAGCAATAG	48
mtSSU-588-5’	588–607	TGTATAGCAATAGCTGATTG	47
mtSSU-1348-3’	1329–1348	CAACGCTTGTAAATATAYTATC	45–47
nuMCM7-790-5’	790–809	TGACYGCRAAGCARTTCAC	52–60
nuMCM7-812-5’	812–830	CATTGACYGAATGYCCTTC	49–56
nuMCM7-1217-3’	1195–1217	GACARGTACTCRTACATRTGRCC	51–61
RPB2-1262-5’	1262–1282	TYCAYAARCTGACCAARGACG	51–62
RPB2-1588-5’	1588–1606	GGYCARGCTTGTGGTCTRG	54–64
RPB2-1261-5’	1261–1281	TTYCAYAARCTGACCAARGAC	49–59
RPB2-2143-3’	2122–2143	GTTGTGARATCAAYAARTCYTC	46–55
RPB2-2138-3’	2117–2138	GAGATCAAYAAGTCYTCKGGAG	51–59
RPB2-1844-3’	1823–1844	AAGCTRACTTCGTGAGARATCA	52–57
nuSSU-1787-5’	1787-end	GAAGGATCATTAWTGAGTKYG	46–52
nuSSU-1785-5’	1785-end	CGGAAGGATCATTAAYGAGT	50–53
nuLSU-21-3’	2–21	CTACCTGATCCGAGGTCAAT	55

Locations of the newly designed primers were determined using reference sequences from various fungal taxa, as listed below. The multiple sequence alignments for all loci are provided in Suppl. materials 1–4, which contain a broad sampling of sequences representing the major *Cladonia* (sub)clades recognized in Stenroos et al. (2019), along with reference primers designed for *Cladonia* taxa. For mtSSU primer locations, the mitochondrial small subunit rRNA reference sequence from *Triangularia anserina* (synonym *Podospora anserina*; GenBank accession number X14734) was used. To improve specificity, the dataset was expanded to include additional environmental ascomycete sequences, but basidiomycete mtSSU sequences hardly align with lichen-forming fungi.

**Figure 1.**
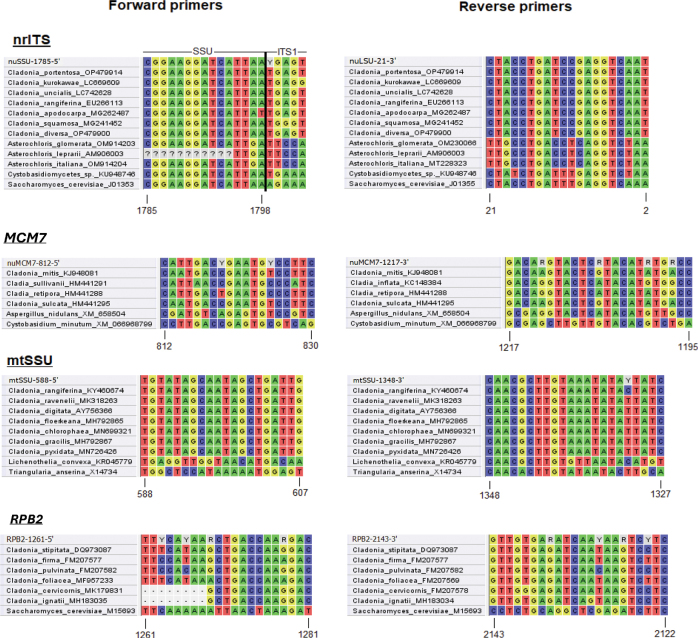
Sequence alignments at primer binding locations. Numbering of nucleotide sites was determined using reference sequences from various fungal taxa. Alignments used for primer design are provided in Suppl. materials 1–4, including representative sequences from major *Cladonia* (sub)clades (Stenroos et al. 2019) and previously published primers.

The *MCM7* primer positions follow the reference sequence from *Aspergillus nidulans* (GenBank accession number XM_658504) (Schmitt et al. 2009). The available sequence of *Cystobasidium minutum* (GenBank accession number XM_066968799) was included to assess potential discrimination against basidiomycete yeasts (Černajová and Škaloud 2019). For *RPB2* primers, the *Saccharomyces cerevisiae* RNA polymerase II, 140 kDa subunit gene (*RPB2*), complete coding sequence (GenBank accession number M15693, YSCPOL2R) was used. The nrITS primer positions within the nuclear rRNA loci (nuSSU and nuLSU) are based on alignments with two reference sequences (GenBank accession numbers J01355 and J01353, M27607). The reverse primer aligns to J01355, whereas the forward primers align to J01353. The forward primer partially overlaps with the ITS1 region at its 3’ end, targeting highly variable sequences for improved taxonomic specificity. This specificity is supported by comparative analysis of priming locations across basidiomycete yeasts, *Saccharomyces* fungi, and *Trebouxia* photobionts, as shown in Fig. 1.

The melting temperature (Tm) of the primers was determined using the NEB Tm Calculator (https://tmcalculator.neb.com/) with parameters characterized for Taq DNA polymerase. For primers containing degenerate bases, Tm values are presented as intervals to reflect the range of possible sequence variants. All primers used in this study showed no evidence of stable self-dimer formation, with the lowest self-dimer Tm being 26 °C as estimated by Geneious Prime® software, indicating low stability (Dieffenbach et al. 1993). Thus, any potential self-dimer formation is unlikely to interfere with the PCR process.

### ﻿Sampling

This study analyzed a collection of 110 specimens from a broad range of species across the genus (Suppl. material 5: *Cladonia* samples). The groupings were determined by nrITS sequencing and divided into clades and subclades within the genus *Cladonia* as indicated by Stenroos et al. (2019) and Pino-Bodas et al. (2024). The collection includes samples from the following clades and subclades: clade *Arbuscula* (7), clade *Impexae* (2), clade *Unciales* (8), subclade *Subglaucescentes* (9), subclade *Ascyphiferae* (6), subclade *Gracilis* (26), subclade *Foliacea* (34), subclade *Helopodium* (2), clade *Crustaceae* (2), and clade *Perviae* (1). Subclade *Subglaucescentes* is part of clade *Erythrocarpae*, whereas the subclades *Ascyphiferae*, *Gracilis*, *Foliacea*, and *Helopodium* all fall within clade *Cladonia*. For the remaining 13 samples, species identification was not possible due to ambiguous results from NCBI BLAST (multiple species listed), unsuccessful PCR amplification, or failed sequencing. These specimens were collected from various locations worldwide, including Iceland, China, Canada, Denmark, Italy, Norway, Hungary, North Macedonia, Albania, and Slovakia.

### ﻿DNA extraction and PCR amplification

The current study included *Cladonia* herbarium specimens collected between 2010 and 2024, most of which were identified using fungal nrITS DNA barcoding. Prior to DNA extraction, lichen thalli were cleaned of any debris. A total of 15–20 mg of lichen tissue was used for whole genomic DNA extraction, following the CTAB method (Cubero et al. 1999). DNA concentration was then measured using Thermo Scientific NanoDrop1000, and only samples with a concentration of ≥ 10 ng/µl were used for PCR amplification.

PCR amplifications were performed in 25 μL reactions containing 1× standard Taq buffer (New England Biolabs), 0.5 μL of 10 mM dNTPs, 0.5 μL of each primer (10 μM), 1 μL of template DNA, and PCR-grade water to reach the final volume. Reactions were run on an Applied Biosystems Veriti™ 96-Well Thermal Cycler (Thermo Fisher Scientific). The thermal profile followed a touchdown strategy adapted from a previously published approach (Xu et al. 2020). The program began with an initial denaturation at 94 °C for 3 minutes, followed by 10 touchdown cycles of 94 °C for 40 seconds, annealing for 35 seconds starting at the primer Tm and decreasing by 1 °C per cycle, and extension at 68 °C for 40 seconds. This was followed by 30 cycles with a fixed annealing temperature of the primer, using the same denaturation and extension steps. A final extension was carried out at 68 °C for 5 minutes. Primer-specific annealing temperatures were selected based on Tm predictions (Table 1), and all annealing steps were kept above 50 °C to ensure primer specificity. Amplicon presence and size were verified by 2% agarose gel electrophoresis using SYBR Safe stain (Invitrogen, CA, USA). Successfully amplified amplicons were purified using ExoSAP (Fermentas Inc., Hanover, MD, USA) and sequenced in both directions via Sanger sequencing (Macrogen Europe BV, the Netherlands) using the same primers as in the PCR amplification.

### ﻿DNA sequence processing

Raw sequence reads were trimmed at both ends to remove ambiguous bases using Geneious Prime® 2024.0.5, and contigs were assembled from forward and reverse strands. Ambiguous base calls were manually reviewed and corrected. The success rates were calculated by dividing the number of specimens with successful PCR amplification by the total number of specimens tested. The multiple sequence alignments were performed using global alignment with a 65% similarity cost matrix in Geneious Prime® 2024.0.5.

### ﻿Statistical analysis

To compare the performance of each genetic marker used in this study, results from successfully amplified samples were evaluated. Site variability (%) was read from multisequence alignments generated in Geneious (Suppl. materials 6–9), reflecting the percentage of variable sites within each marker alignment. Nucleotide diversity (π) was calculated using the package *pegas* (Paradis 2010) in R (R Core Team 2024) using RStudio (Version 2024.12.0+467), with sequence alignments for each marker.

To evaluate the relative informativeness of each marker, we analyzed pairwise genetic distances among nrITS, *RPB2*, *MCM7*, and mtSSU. A dataset of 36 *Cladonia* samples that produced high-quality sequences for all four loci was selected to ensure comparability across all markers. Sequence alignments for nrITS, *RPB2*, *MCM7*, and mtSSU were generated using MAFFT v7. Pairwise distances were calculated in the R package ape (Paradis and Schliep 2019) under the Kimura 2-parameter (K80) model. Distances were assigned to three categories: within subclade, between subclades within the same clade, and between clades. Distance data were summarized as the number of comparisons, mean, median, and standard deviation. Boxplots were created in *ggplot2* (Wickham 2016) to visualize the distributions and allow direct comparison. To statistically evaluate differences between categories, Wilcoxon rank-sum tests were conducted for comparison of within vs. between subclades and between subclades vs. between clades.

## ﻿Results

### ﻿Primer design

The priming locations within the fungal reference sequences for the newly designed primers (Table 1) are shown in Fig. 1. The fungal nrITS forward primer spans the nuSSU and ITS1 regions, whereas the reverse primer is fully located within the nuLSU region. Both primers are designed to anneal to the lichen-forming fungi while discriminating against the photobionts and basidiomycete yeasts. The *MCM7* primers have limited amplicon sizes but still distinguish against basidiomycetes. The newly designed mtSSU primers target conserved regions within Lecanoromycetes, which are highly divergent in other fungal groups, contributing to their high specificity for lichen-forming fungi. The high nucleotide variation in the *RPB2* marker makes it more challenging to design specific primers.

### ﻿PCR amplification success

**Figure 2.**
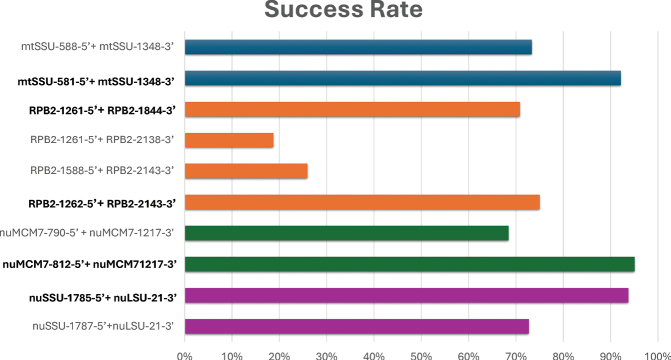
Primer pair amplification success rate. The bar chart displays the percentage of samples successfully amplified by each primer pair. Primer pairs with the highest amplification success rates are highlighted in bold.

For each of the ten primer pairs used across the four target loci, the PCR amplification success was calculated. The combined results are presented in Fig. 2.

Both mtSSU primer pairs had high amplification success rates. The *RPB2* primer pairs showed clear differences in amplification success. The RPB2-1588-5’ + RPB2-2143-3’ and RPB2-1261-5’ + RPB2-2138-3’ primer pairs showed relatively low success rates. However, the RPB2-1262-5’ + RPB2-2143-3’ and RPB2-1261-5’ + RPB2-1844-3’ primers had significantly higher success rates of 75% and 70%, respectively. For the *MCM7* locus, the success rate for the nuMCM7-812-5’ + nuMCM7-1217-3’ primer pair was 95%, whereas the nuMCM7-790-5’ + nuMCM7-1217-3’ primer pair had a lower success rate of 68%. The nrITS primer pairs both worked well, and the highest success rate was produced by the nuSSU-1785-5’ + nuLSU-21-3’ primer pair with 93%.

### ﻿Sequence quality

**Figure 3.**
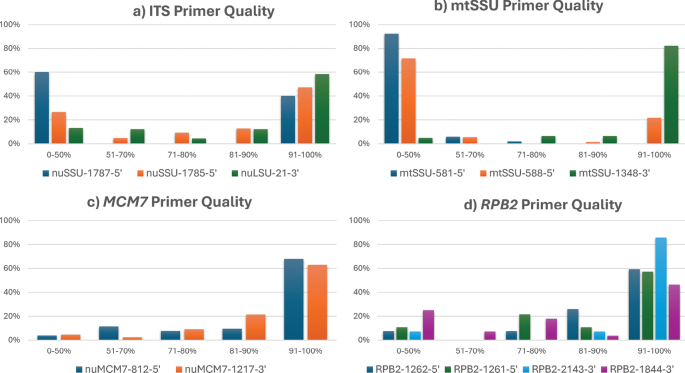
Primer percent high-quality values (% HQ). The Y-axis represents the percentage of all samples, whereas the X-axis shows the intervals into which each sequence falls based on its % HQ value. The data are categorized into five intervals: < 50%, 51–70%, 71–80%, 81–90%, and 91–100%. a. nrITS primers; b. mtSSU primers; c. *MCM7* primers; d. *RPB2* primers.

Percent high-quality bases (% HQ) were used to assess the quality of the sequenced data. This measure represents the percentage of bases in the sequence data that meet specific quality thresholds. All samples were trimmed with a 0.05 error probability limit and a maximum of 2 ambiguities at the 5’ and 3’ ends. After trimming, the % HQ values were recorded and compared for each primer; the results are shown in Fig. 3.

A % HQ of > 90% is considered ideal, ensuring reliable and accurate results. Values between 80% and 90% are generally acceptable, while 70–80% indicate minor quality issues. Sequences within the 50–70% range require additional quality control, and those below 50% may compromise the analysis. For sequences between 50% and 80%, an alignment with higher-quality complementary strands was performed and corrected for accuracy. Only sequences with good alignment matches were used for further analysis. This approach ensured that data integrity was maintained for downstream analyses.

The nrITS forward and reverse primers produced sequences with variable % HQ values (Fig. 3a). The primer nuSSU-1787-5’ mostly generated sequences with poor quality, as 60% of samples fell within the 0–50% interval. nuSSU-1785-5’ had better performance, with 47% of sequences in the 90–100% interval. The results from nuLSU-21-3’ sequences showed the reliability of the reverse primer in generating high-quality sequences.

The sequence quality from mtSSU primers was similarly variable (Fig. 3b). The mtSSU-581-5’ primer exhibited poor sequencing quality, with 92% of samples producing unusable results and showing numerous heterozygous peaks. In contrast, mtSSU-588-5’ demonstrated improved results, with more samples in the 91–100% interval. A large portion of the 0–50% interval results for mtSSU-588-5’ originated from PCR products amplified by mtSSU-581-5’ and sequenced with the new mtSSU-588-5’ primer. A potential second binding site for the mtSSU-581-5’ primer, located 154 bp earlier, could explain the observed heterozygous peaks. The mtSSU-1348-3’ primer consistently generated high-quality sequence data, with 80% of samples in the 91–100% interval. These high-quality reverse-strand sequences were instrumental in correcting the lower-quality forward-strand sequences, ensuring reliable and accurate data for further analyses. Although sequencing inconsistencies were caused by the forward primer, the consistently high-quality sequences from the reverse primer indicate that PCR amplification was successful.

The forward primer nuMCM7-812-5’ performed well, with 68% of sequences in the 91–100% interval, and the reverse primer showed similarly high reliability (Fig. 3c). Sequences that were amplified with nuMCM7-790-5’+ nuMCM7-1217-3’ were identical to those obtained with nuMCM7-812-5′, so they were excluded from further analysis.

Comparing the *RPB2* forward primers in Fig. 3d, RPB2-1262-5’ and RPB2-1261-5’ generated similar numbers of sequences in the 91–100% range. However, RPB2-1262-5’ had more sequences in the 81–90% interval, whereas RPB2-1261-5’ had more in the 71–80% range, indicating slightly lower quality. For the reverse primers, RPB2-2143-3’ demonstrated excellent performance, while RPB2-1844-3’ had a higher proportion of sequences in the lower intervals.

### ﻿Literature comparison

To evaluate the relative performance of our primers, the observed amplification success rates were compared with those reported for existing primer sets targeting nrITS, *RPB2*, *MCM7*, and mtSSU in *Cladonia* and other lichenized fungi. Reported values vary widely among studies, ranging from < 50% for some *RPB2* primer pairs (Yahr et al. 2006; Stenroos et al. 2019) to > 90% for commonly used nrITS and mtSSU primers (Zoller et al. 1999; Piercey-Normore and DePriest 2001). Success rates for *MCM7* primers have generally been moderate to high (70–85%), but with limitations in sequence length (Schmitt et al. 2009; Lumbsch et al. 2010). Our newly designed primer sets consistently performed at or above the upper range of these values, particularly for *RPB2*, where amplification success exceeded most previous reports. A summary of literature values, together with their reported limitations, is presented in Table 2.

**Table 2.** Summary of previously published primer sets for nrITS, *RPB2*, *MCM7*, and mtSSU in *Cladonia* and related lichenized fungi. Reported PCR success and sequencing performance are listed alongside limitations noted by the authors.

Primers	Study	Reported PCR success	Reported limitations
SSU-1780 + LSU-0012 (*Cladonia*-specific primers)	Piercey-Normore and DePriest 2001	> 90% in multiple *Cladonia* spp.	Risk of co-amplifying photobiont DNA
ITS1F + ITS4 (fungal “universal” primers)	Gardes and Bruns 1993	Generally high success	Non-specific binding, algal/contaminant DNA
SSU-1780 + ITS4	Yahr et al. 2006	Moderate success in *Cladonia subtenuis* complex	Co-amplification of algal partners; variable sequencing quality
ITS1F + ITS4 (fungal “universal” primers)	Pino-Bodas et al. 2013	85–95% success in herbarium and fresh specimen	Incomplete species resolution; frequent intragenomic variation
fRPB2-5F + fRPB2-7cR (general fungal primers)	Liu et al. 1999	50–80% across Ascomycota	Introns cause amplification failure; long amplicon not herbarium-friendly
CLRPB5F + CLRPB7R (*Cladonia*-specific primers)	Yahr et al. 2006	40–60% success	Poor performance in degraded herbarium DNA; frequent failure
RPB2dRaq/RPB2rRaq (*Cladonia*-specific primers)	Pino-Bodas et al. 2010	50–70% success depending on taxon	High rate of no-product amplifications; alignment difficulty across introns
Various general *RPB2* primers	Stenroos et al. 2019	< 50% success in some lineages	Difficult amplification; poor sequencing from degraded DNA
Various general *RPB2* primers	Reeb et al. 2004	50–80% success across ascomycetes	Not optimized for lichen fungi; variable across clades
Mcm7-709for + Mcm7-1348rev (general primers)	Schmitt et al. 2009	70–85% across lichenized Ascomycota	Short locus, sometimes limited phylogenetic signal
MS456 + MS277 (degenerate primers)	Schmitt et al. 2009	96% success across 44/46 taxa	Degeneracy leads to potential non-specific binding; may need optimization
Mcm7-709for + Mcm7-1348rev (general primers)	Raja et al. 2011	~85% in Ascomycota	Mixed amplification success in lichens; some sequencing difficulties
Mcm7-709for + Mcm7-1348rev (general primers)	Lumbsch et al. 2010	Moderate success	Sequences sometimes too short to resolve species-level relationships
mrSSU1 + mrSSU3R	Zoller et al. 1999	> 90% success in lichens including *Cladonia*	Highly conserved; limited for recent divergences
mtSSU1 + mtSSU3R	Zhou and Stanosz 2001	80–95%	Some primer mismatch across taxa; occasional off-targets
mtSSU-581-5’ + mtSSU-1345-3’	Xu et al. 2023	~ 80% in *Melanelia* (closely related genus)	Off-target amplification in herbarium material; lower quality sequences
Various general mtSSU primers	Stenroos et al. 2019	> 85% success overall	Limited phylogenetic resolution; often excluded from fine-scale analyses

### ﻿BLAST results

BLAST results were used for specimen identification using the nrITS locus. The sequence data were matched by aligning forward and reverse reads, which were manually corrected and further trimmed for accuracy. These curated sequences were then run through BLAST, with grade percentage (sequence identity) as a critical metric for alignment accuracy. A 97% identity threshold is widely used for species-level identification of fungi using nrITS sequences. Percent identities above 97% generally indicate strong species-level matches, whereas lower values indicate more distant relationships (Nilsson et al. 2008; Bradshaw et al. 2023). The nrITS results confirmed that most samples are a *Cladonia* species, with only two samples matching uncultured fungi species (CLX37, CLX44). In addition to nrITS, the *RPB2* locus was also tested for its application in specimen identification.

**Figure 4.**
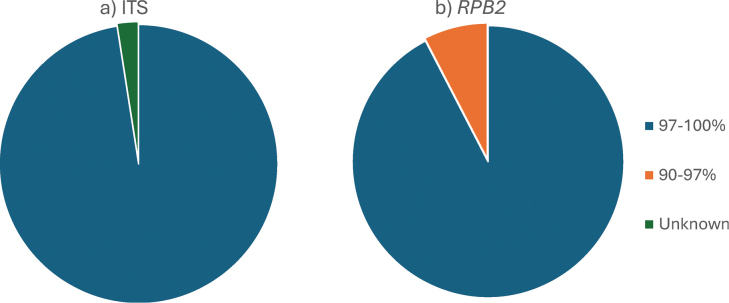
BLAST results identifying *Cladonia* specimens, represented in pie charts. In each chart, blue indicates samples with high-confidence matches (≥ 97%), orange represents moderate-confidence matches (90–97%), and green shows samples that could not be identified using BLAST. a. Results from nrITS amplicons; b. Results from *RPB2* amplicons.

Fig. 4 provides a summary of the BLAST results. A total of 81 nrITS sequences were analyzed using BLAST, with 79 samples achieving a grade percentage above 97% and two identified as uncultured fungi. A total of 105 *RPB2* sequences were analyzed, and 97 achieved a species-level match. The results were generally strong, though many samples received equally high grades for multiple *Cladonia* species, making species-level identification challenging. However, it was still possible to group many samples into their respective clades and subclades.

A total of 77 samples were analyzed using both nrITS and *RPB2* markers (Suppl. material 10). Of these, 53 samples matched at the clade or subclade level. However, species-level identification showed more variation, with only 20 samples receiving the same species designation as the top BLAST hit for both loci. This discrepancy highlights potential differences in resolution between the markers.

nrITS sequences from the 77 samples matched 27 different *Cladonia* species, whereas *RPB2* sequences from the same set of samples matched 19 species. Although *RPB2* has been proposed as an additional barcoding gene alongside ITS for species-level identification, our results do not support this use. The reduced species recovery for *RPB2* likely reflects both its lower variability compared to ITS and the limited representation of *RPB2* sequences in GenBank.

The availability of nrITS and *RPB2* sequences for *Cladonia* was also compared using a BLAST search of the NCBI Nucleotide database. This search yielded 3,911 hits for nrITS sequences, reflecting the widespread use of the locus in fungal barcoding and species identification. In contrast, the search for *RPB2* sequences returned only 459 hits, indicating that this locus is less commonly sequenced in *Cladonia* species.

### ﻿Marker evaluation and genetic distance distribution

To provide an overview of marker performance, this summary incorporates data from all samples and all primer pairs used in the study. It reflects the overall performance of each genetic marker by reporting the output of usable amplicons obtained, sequence length, and the resulting variability across all tested combinations (Table 3).

**Table 3.** Summary statistics for each genetic marker, including number of sequences, average length of sequences (bp), site variability (%), and nucleotide diversity (π), across all sampled taxa.

Marker	Number of sequences	Sequence length average (bp)	Site variability (%)	Nucleotide diversity (π)
**nrITS**	99	624	51%	0.044
**mtSSU**	43	630	14%	0.010
***MCM7***	87	377	36%	0.032
***RPB2***	81	824	35%	0.043

A total of 310 sequences were obtained across the four genetic markers. The number of sequences per marker ranged from 43 (mtSSU) to 99 (nrITS), with average sequence lengths varying between 377 bp (*MCM7*) and 824 bp (*RPB2*). nrITS exhibited the highest site variability (51%) and nucleotide diversity (π = 0.044), indicating that it was the most variable marker. In contrast, mtSSU showed the lowest variability and diversity, suggesting limited resolution for fine-scale genetic differentiation.

To evaluate how genetic variation is structured across the dataset, 36 nrITS sequences were grouped into clade *Arbuscula*, clade *Crustaceae*, clade *Impexae*, clade *Perviae*, clade *Unciales*, subclade *Ascyphiferae*, subclade *Foliaceae*, subclade *Graciles*, subclade *Subglaucescentes*, and subclade *Helopodium* (Table 4).

**Table 4.** BLAST-based classification of nrITS sequences in groups of *Cladonia* by clades and subclades.

Grouping	Samples
**clade *Arbuscula***	CLX3, CR1
**clade *Crustaceae***	CLX22, CLX31
**clade *Impexae***	CLX30, CLX43
**clade *Perviae***	CLX48
**clade *Uncialis***	CLX2
**subclade *Ascyphiferae* (clade *Cladonia*)**	CLX21, CLX33, CLX38, CLX39
**subclade *Foliaceae* (clade *Cladonia*)**	CLF10, CLF24, CLX24, CLX25, CLX26
**subclade *Graciles* (clade *Cladonia*)**	CLI1, CLI2, CLI3, CLX8, CLX16, CLX17, CLX23, CLX35, CLX36, CLX47, CP1, CY1
**subclade *Helopodium* (clade *Cladonia*)**	CLX40
**subclade *Subglaucescentes* (clade *Erythrocarpae*)**	CB1, CB5, CLX13b, CLX27, CLX29, CLX34

**Figure 5.**
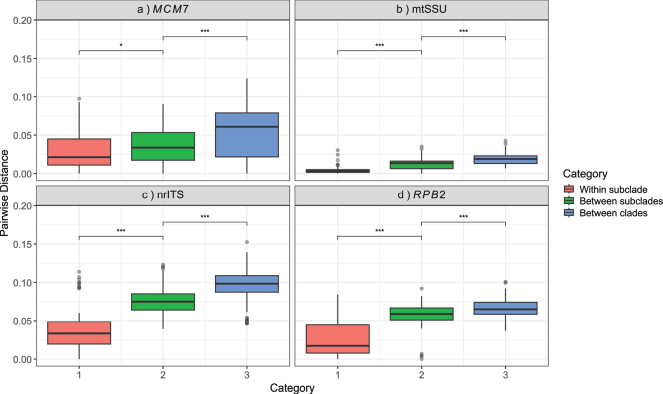
Boxplots of pairwise distances for each marker, separated by category (within subclade, between subclades, between clades). Analyses were conducted for all four markers: a. *MCM7*; b. mtSSU; c. nrITS; d. *RPB2*. Asterisks above horizontal lines represent p-values from Wilcoxon rank-sum tests between adjacent categories are displayed above the boxes to indicate significant differences: *: 0.01 < p <= 0.05; ***: p < 0.001.

To characterize the informativeness of the four markers, the distributions of pairwise genetic distances (Suppl. materials 11–14) were examined across hierarchical levels of divergence. The increase in pairwise distances from within subclades to between clades across all four markers is shown in Table 5 and visualized in boxplots in Fig. 5.

**Table 5.** Summary of pairwise genetic distances for each marker in the categories (1) within subclades, (2) between subclades within the same clade, and (3) between clades. Values include the number of comparisons (n), mean, median, standard deviation (SD), and Wilcoxon test p-values for each category.

Marker	Category	n	Mean	Median	SD	P-value
**nrITS**	1	84	0.0378	0.0334	0.0282	< 0.001
	2	123	0.0746	0.0750	0.0181	< 0.001
	3	289	0.0972	0.0984	0.0183	< 0.001
***MCM7***	1	84	0.0255	0.0172	0.0230	0.0399
	2	123	0.0577	0.0589	0.0144	0.0399
	3	289	0.0668	0.0651	0.0116	< 0.001
***RPB2***	1	84	0.00417	0.00325	0.00490	< 0.001
	2	123	0.0125	0.0135	0.00677	< 0.001
	3	289	0.0183	0.0189	0.00655	< 0.001
**mtSSU**	1	84	0.0291	0.0213	0.0242	< 0.001
	2	123	0.0352	0.0336	0.0239	< 0.001
	3	289	0.0522	0.0608	0.0329	< 0.001

ITS displayed the greatest variability, with clear separation among the three categories. Wilcoxon tests indicated highly significant differences for both within vs. between subclades and between subclades vs. between clades. *RPB2* displayed intermediate divergence, with statistically significant separation between adjacent categories. *MCM7* also showed intermediate distances, but the separation between within and between subclades was less pronounced. This is reflected in a marginally significant Wilcoxon test (p = 0.0399), suggesting that *MCM7* may have slightly lower resolution at the subclade level compared to ITS or *RPB2*. The separation between between-subclades and between-clades remained highly significant (p < 0.001). mtSSU exhibited the lowest distances, reflecting its highly conserved nature, yet still showed significant separation between categories.

## ﻿Discussion

This study presents newly designed primer sets for four widely used genetic loci in mycobiont research. For the nrITS marker, although the reverse primer binds to a highly variable 3’ region, the forward primer was specifically designed to target a *Cladonia*-specific region to improve amplification specificity. The forward primers partially overlap with the published primer nrSSU-1780 (Piercey-Normore and DePriest 2001) but are extended by one to three nucleotides to increase selectivity for *Cladonia* fungi. Designing primers for the *MCM7* locus was challenging due to the limited number of reference sequences (Lumbsch et al. 2010), making it difficult to design primers for longer amplicons. The primers amplifying the short amplicons (~380 bp) performed well across the *Cladonia* genus. Primers for the mtSSU region were improved by placing the forward primer between the conserved regions of U5 and U6 (Xu et al. 2023).

The high nucleotide variation in the *RPB2* locus presents challenges for primer design. During alignment construction, the first half of the ca. 900 bp-long amplicons was observed to be more variable than the second half, which led to a primer design that targeted the more informative region. High amplification success is generally defined as achieving at least 80% amplification across taxa, a threshold met by widely used fungal primers for nuSSU, nuLSU, and nrITS loci. In contrast, protein-coding genes such as *RPB2* tend to exhibit lower success rates, ranging from 50% to 80%, due to intron variation and sequence divergence (Reeb et al. 2004). Based on both sequence alignment and PCR performance, our new *RPB2* primers show good specificity for *Cladonia* fungi and outperform previously published primers used for phylogenetic analyses (Liu et al. 1999; Yahr et al. 2006; Pino-Bodas et al. 2010; Stenroos et al. 2019). Although efficiency varied slightly, both *MCM7* primer sets together enabled full sample coverage. The nrITS and mtSSU primers also demonstrated strong amplification success. *RPB2* primers had variable success rates, and the low success rates observed in some pairs (e.g., RPB2-1261-5’ + RPB2-2138-3’) were likely due to primer–template mismatches or inefficient binding (Ghedira et al. 2009). These results are above the expected success range and further highlight the importance of primer selection for each marker and taxonomic group.

While most sequences were of high quality, variation in primer performance emphasized the importance of evaluating both primers in a pair to ensure sequence reliability. For the nrITS region, nuSSU-1787-5’ often produced low-quality sequences, aligning with known amplification challenges (Raja et al. 2011), whereas nuSSU-1785-5’ performed better, likely due to improved specificity (Bustin and Huggett 2017). The reverse primer nuLSU-21-3’ consistently enhanced overall sequence quality. In the mtSSU region, the mtSSU-581-5’ primer performed poorly, likely due to secondary binding sites. Although the alternative forward primer (mtSSU-588-5’) showed improvement in sequencing quality, the reverse primer (mtSSU-1348-3’) was particularly valuable for correcting poor-quality reads by consistently producing high-quality sequences. Instances of reduced sequence quality are best explained by primer annealing issues or secondary binding sites, as alternative primers improved results on the same extracts. The *MCM7* primer set produced high-quality sequences across the sample set, indicating good primer design and target compatibility. For *RPB2*, both forward primers were effective, whereas the reverse primer RPB2-2143-3’ was more reliable than RPB2-1844-3′.

Comparison with previously published primer sets indicates that the newly designed primers achieve equal or higher amplification success while reducing common issues such as algal co-amplification and consistently producing high-quality sequences across a broad collection of *Cladonia* species.

BLAST results generally supported clade-level groupings but provided limited species-level resolution for *Cladonia*. Species identification faced two major challenges. First, there were discrepancies among markers likely stemming from the significantly greater number of nrITS sequences available for *Cladonia* compared to *RPB2*. Second, multiple species matches in the nrITS results likely reflect overlapping genetic signals, incomplete lineage sorting, and insufficient barcode gaps (Pino-Bodas et al. 2013; Stenroos et al. 2019; Steinová et al. 2022). These limitations are consistent with findings that nrITS often fails to distinguish morphologically distinct but genetically similar *Cladonia* species (Kanz et al. 2015). The two nrITS sequences matching uncultured fungi suggest off-target amplification or limited database coverage. *RPB2* improved resolution in some cases but also showed inconsistencies, likely due to lower marker variability and sparse sequence representation. These results highlight the importance of multi-locus approaches, careful marker selection, and taxon sampling for reliable molecular identification of lichenized fungi (Větrovský et al. 2016).

All markers showed high PCR success rates, confirming their technical reliability across a broad range of *Cladonia* taxa, yet their ability to resolve genetic structure differed markedly. nrITS and *RPB2* emerged as the most informative loci; nrITS exhibited the highest sequence variability, making it particularly effective for distinguishing closely related species and subclades, whereas *RPB2* displayed intermediate divergence suitable for resolving relationships at moderate evolutionary depths. *MCM7* showed intermediate divergence as well, with slightly lower resolution at the subclade level than the other markers but strong informativeness at the clade level, whereas mtSSU was highly conserved and primarily captured deeper phylogenetic splits (Kanz et al. 2015). The consistently high PCR success and sequence quality, particularly for *MCM7*, ensured robust data, although its short amplicon length (~377 bp) likely constrained resolution at finer taxonomic scales (Min and Hickey 2007). Future efforts should prioritize extending amplicon length, improving reference databases, and expanding sampling within underrepresented subclades to enhance phylogenetic resolution and species-level discrimination. These results highlight that the resolution of genetic structure depends on the inherent variability of each marker and that combining multiple loci with complementary evolutionary rates provides a stronger framework for phylogenetic inference and species delimitation in *Cladonia*.

## ﻿Conclusion

This study presents new mycobiont-specific primers for nrITS, mtSSU, *MCM7*, and *RPB2* that provide reliable amplification and high-quality sequence data across a broad range of *Cladonia* taxa. Each locus contributes complementary information: nrITS is most informative for distinguishing closely related species and subclades, *RPB2* provides intermediate resolution for moderate evolutionary depths, *MCM7* offers high-quality sequences suitable for clade-level analyses, and mtSSU is best suited for capturing deeper phylogenetic splits. Based on these results, we recommend nrITS as the primary locus for species-level identification, with *RPB2* and *MCM7* as complementary markers for phylogenetic studies and mtSSU for resolving deeper divergences. These mycobiont-specific primers will be useful for future multi-locus studies in *Cladonia*, and in combination they may provide increased resolution and improved taxonomic coverage.

## ﻿Additional information

### Use of AI

AI tools were used to review and check text for language refinement and organization of text. All intellectual contributions, data interpretation and conclusion were original work from the authors.

### Author ORCIDs

Maia Biwersi https://orcid.org/0009-0007-8072-9430

Maonian Xu https://orcid.org/0000-0002-4346-8020

Starri Heiðmarsson https://orcid.org/0000-0003-0329-6882

Snæbjörn Pálsson https://orcid.org/0000-0002-4297-3500

John L. Sorensen https://orcid.org/0000-0002-1648-2310

Elín S. Ólafsdóttir https://orcid.org/0000-0001-7305-7129

### Data availability

All of the data that support the findings of this study are available in the main text or Supplementary Information.

## Supplementary materials

Supplementary material 1 Primer alignment nrITS Data type fas Explanation note Primer sequences aligned to reference sequence to show the primer binding locations for the genetic marker nrITS. https://binary.pensoft.net/file/1506461This dataset is made available under the Open Database License (http://opendatacommons.org/licenses/odbl/1.0/). The Open Database License (ODbL) is a license agreement intended to allow users to freely share, modify, and use this Dataset while maintaining this same freedom for others, provided that the original source and author(s) are credited.Maia Biwersi, Maonian Xu, Starri Heiðmarsson, Snæbjörn Pálsson, John L. Sorensen, Elín S. Ólafsdóttir

Supplementary material 2 Primer alignment *MCM7* Data type fas Explanation note Primer sequences aligned to reference sequence to show the primer binding locations for the marker gene *MCM7*. https://binary.pensoft.net/file/1506462This dataset is made available under the Open Database License (http://opendatacommons.org/licenses/odbl/1.0/). The Open Database License (ODbL) is a license agreement intended to allow users to freely share, modify, and use this Dataset while maintaining this same freedom for others, provided that the original source and author(s) are credited.Maia Biwersi, Maonian Xu, Starri Heiðmarsson, Snæbjörn Pálsson, John L. Sorensen, Elín S. Ólafsdóttir

Supplementary material 3 Primer alignment mtSSU Data type fas Explanation note Primer sequences aligned to reference sequence to show the primer binding locations for marker gene mtSSU. https://binary.pensoft.net/file/1506463This dataset is made available under the Open Database License (http://opendatacommons.org/licenses/odbl/1.0/). The Open Database License (ODbL) is a license agreement intended to allow users to freely share, modify, and use this Dataset while maintaining this same freedom for others, provided that the original source and author(s) are credited.Maia Biwersi, Maonian Xu, Starri Heiðmarsson, Snæbjörn Pálsson, John L. Sorensen, Elín S. Ólafsdóttir

Supplementary material 4 Primer alignment *RPB2* Data type fas Explanation note Primer sequences aligned to reference sequence to show the primer binding locations for the marker gene *RPB2*. https://binary.pensoft.net/file/1506464This dataset is made available under the Open Database License (http://opendatacommons.org/licenses/odbl/1.0/). The Open Database License (ODbL) is a license agreement intended to allow users to freely share, modify, and use this Dataset while maintaining this same freedom for others, provided that the original source and author(s) are credited.Maia Biwersi, Maonian Xu, Starri Heiðmarsson, Snæbjörn Pálsson, John L. Sorensen, Elín S. Ólafsdóttir

Supplementary material 5 *Cladonia* samples Data type xlsx Explanation note Spreadsheet containing metadata for each *Cladonia* sample used in the study, including collection details, GenBank accession numbers, and subclade assignments. Accession numbers highlighted in the spreadsheet correspond to sequences previously published (Farkas et al. 2024). https://binary.pensoft.net/file/1506465This dataset is made available under the Open Database License (http://opendatacommons.org/licenses/odbl/1.0/). The Open Database License (ODbL) is a license agreement intended to allow users to freely share, modify, and use this Dataset while maintaining this same freedom for others, provided that the original source and author(s) are credited.Maia Biwersi, Maonian Xu, Starri Heiðmarsson, Snæbjörn Pálsson, John L. Sorensen, Elín S. Ólafsdóttir

Supplementary material 6 nrITS alignment Data type fas Explanation note Alignment file of nrITS sequences generated in this study. https://binary.pensoft.net/file/1506466This dataset is made available under the Open Database License (http://opendatacommons.org/licenses/odbl/1.0/). The Open Database License (ODbL) is a license agreement intended to allow users to freely share, modify, and use this Dataset while maintaining this same freedom for others, provided that the original source and author(s) are credited.Maia Biwersi, Maonian Xu, Starri Heiðmarsson, Snæbjörn Pálsson, John L. Sorensen, Elín S. Ólafsdóttir

Supplementary material 7 *MCM7* alignment Data type fas Explanation note Alignment file of *MCM7* sequences generated in this study. https://binary.pensoft.net/file/1506467This dataset is made available under the Open Database License (http://opendatacommons.org/licenses/odbl/1.0/). The Open Database License (ODbL) is a license agreement intended to allow users to freely share, modify, and use this Dataset while maintaining this same freedom for others, provided that the original source and author(s) are credited.Maia Biwersi, Maonian Xu, Starri Heiðmarsson, Snæbjörn Pálsson, John L. Sorensen, Elín S. Ólafsdóttir

Supplementary material 8 Sequence alignments mtSSU Data type fas Explanation note Alignment file of mtSSU sequences generated in this study. https://binary.pensoft.net/file/1506468This dataset is made available under the Open Database License (http://opendatacommons.org/licenses/odbl/1.0/). The Open Database License (ODbL) is a license agreement intended to allow users to freely share, modify, and use this Dataset while maintaining this same freedom for others, provided that the original source and author(s) are credited.Maia Biwersi, Maonian Xu, Starri Heiðmarsson, Snæbjörn Pálsson, John L. Sorensen, Elín S. Ólafsdóttir

Supplementary material 9 Sequence alignments RPB2 Data type fas Explanation note Alignment file of RPB2 sequences generated in this study. https://binary.pensoft.net/file/1506469This dataset is made available under the Open Database License (http://opendatacommons.org/licenses/odbl/1.0/). The Open Database License (ODbL) is a license agreement intended to allow users to freely share, modify, and use this Dataset while maintaining this same freedom for others, provided that the original source and author(s) are credited.Maia Biwersi, Maonian Xu, Starri Heiðmarsson, Snæbjörn Pálsson, John L. Sorensen, Elín S. Ólafsdóttir

Supplementary material 10 BLAST results Data type xlsx Explanation note Spreadsheet containing the top BLAST hit, grade level, and corresponding accession number for nrITS and RPB2 sequences of 77 *Cladonia* samples. The file also reports the identified *Cladonia* species and the subclade/clade assignment for each sample. https://binary.pensoft.net/file/1506470This dataset is made available under the Open Database License (http://opendatacommons.org/licenses/odbl/1.0/). The Open Database License (ODbL) is a license agreement intended to allow users to freely share, modify, and use this Dataset while maintaining this same freedom for others, provided that the original source and author(s) are credited.Maia Biwersi, Maonian Xu, Starri Heiðmarsson, Snæbjörn Pálsson, John L. Sorensen, Elín S. Ólafsdóttir

Supplementary material 11 nrITS distance matrix Data type csv Explanation note Pairwise genetic distance matrix generated from nrITS sequences included in this study. https://binary.pensoft.net/file/1506471This dataset is made available under the Open Database License (http://opendatacommons.org/licenses/odbl/1.0/). The Open Database License (ODbL) is a license agreement intended to allow users to freely share, modify, and use this Dataset while maintaining this same freedom for others, provided that the original source and author(s) are credited.Maia Biwersi, Maonian Xu, Starri Heiðmarsson, Snæbjörn Pálsson, John L. Sorensen, Elín S. Ólafsdóttir

Supplementary material 12 *MCM7* distance matrix Data type csv Explanation note Pairwise genetic distance matrix generated from *MCM7* sequences included in this study. https://binary.pensoft.net/file/1506472This dataset is made available under the Open Database License (http://opendatacommons.org/licenses/odbl/1.0/). The Open Database License (ODbL) is a license agreement intended to allow users to freely share, modify, and use this Dataset while maintaining this same freedom for others, provided that the original source and author(s) are credited.Maia Biwersi, Maonian Xu, Starri Heiðmarsson, Snæbjörn Pálsson, John L. Sorensen, Elín S. Ólafsdóttir

Supplementary material 13 mtSSU distance matrix Data type csv Explanation note Pairwise genetic distance matrix generated from mtSSU sequences included in this study. https://binary.pensoft.net/file/1506473This dataset is made available under the Open Database License (http://opendatacommons.org/licenses/odbl/1.0/). The Open Database License (ODbL) is a license agreement intended to allow users to freely share, modify, and use this Dataset while maintaining this same freedom for others, provided that the original source and author(s) are credited.Maia Biwersi, Maonian Xu, Starri Heiðmarsson, Snæbjörn Pálsson, John L. Sorensen, Elín S. Ólafsdóttir

Supplementary material 14 *RPB2* distance matrix Data type csv Explanation note Pairwise genetic distance matrix generated from *RPB2* sequences included in this study. https://binary.pensoft.net/file/1506474This dataset is made available under the Open Database License (http://opendatacommons.org/licenses/odbl/1.0/). The Open Database License (ODbL) is a license agreement intended to allow users to freely share, modify, and use this Dataset while maintaining this same freedom for others, provided that the original source and author(s) are credited.Maia Biwersi, Maonian Xu, Starri Heiðmarsson, Snæbjörn Pálsson, John L. Sorensen, Elín S. Ólafsdóttir
